# Supplementary figures and images for: Low-Dose and Long-Term Olaparib Treatment Sensitizes MDA-MB-231 and SUM1315 Triple-Negative Breast Cancers Spheroids to Fractioned Radiotherapy
Source: J Clin Med. 2019 Dec 26;9(1):64. doi: 10.3390/jcm9010064 (PMC7019977; doi:10.3390/jcm9010064)

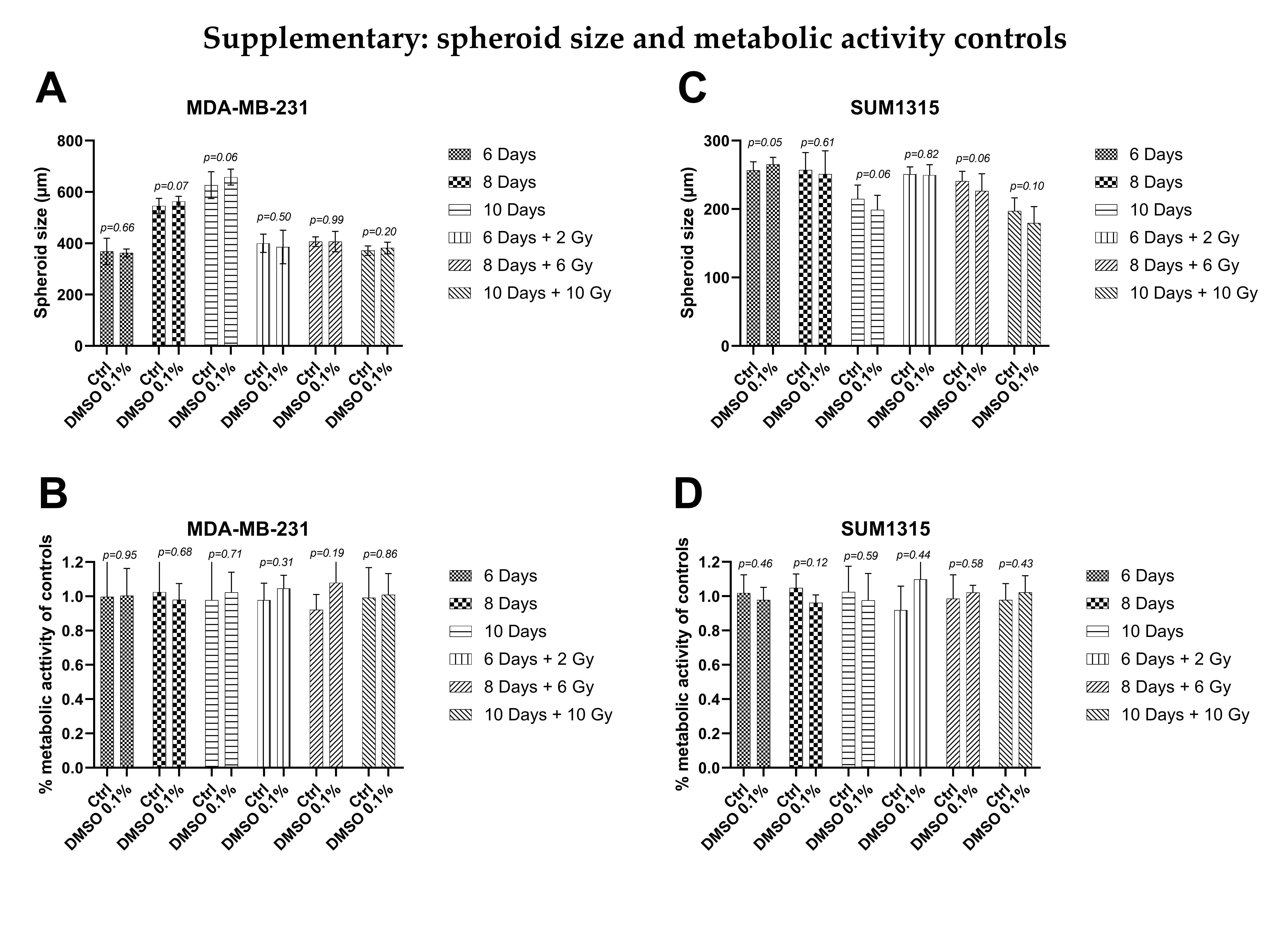

Supplement: Supplementary file 1 [file jcm-09-00064-s001.zip › Supplementary Review/Figure S2.tif]

# Supplementary: 2D cell survival dataset

**A** MDA-MB-231

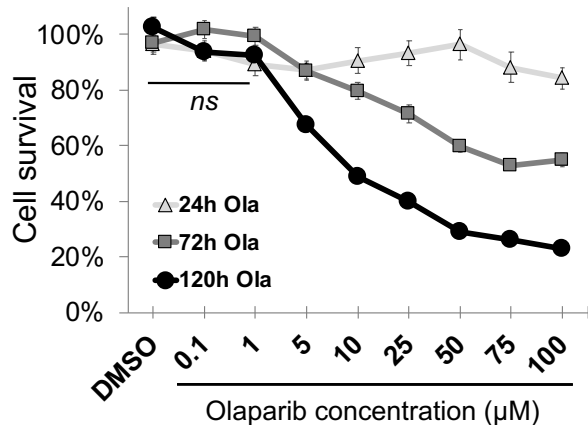

**B** SUM 1315

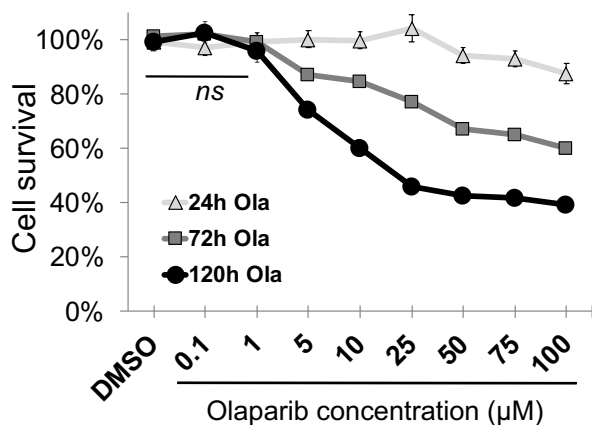

**C**

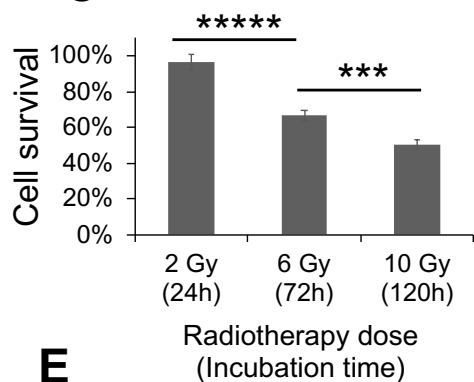

**D**

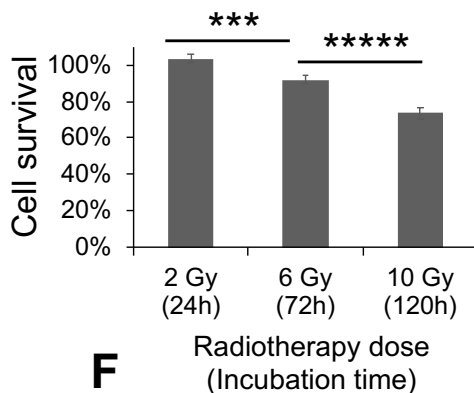

**E**

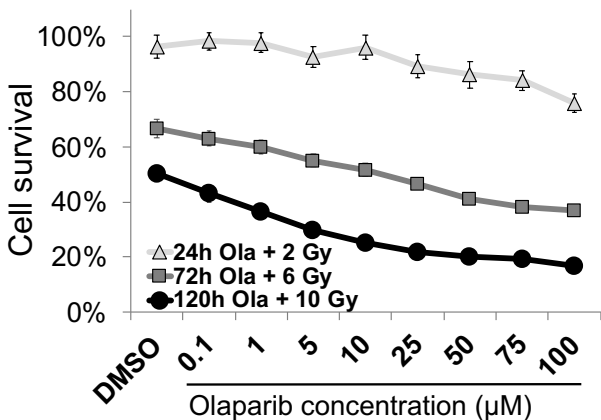

**F**

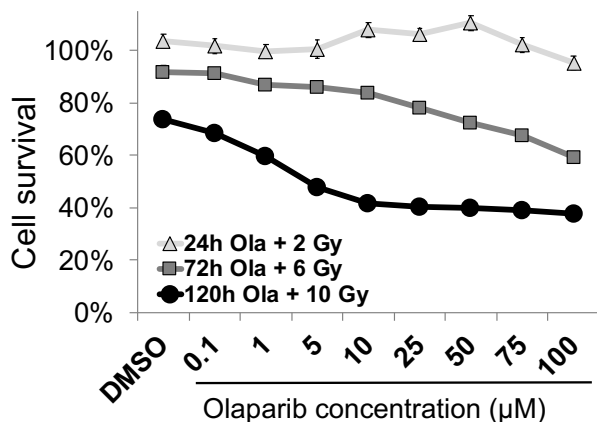

Supplement: Supplementary file 1 [file jcm-09-00064-s001.zip › Supplementary Review/Figure S1.pdf]
